# Emergence of Mobile Colistin Resistance (*mcr-8*) in a Highly Successful Klebsiella pneumoniae Sequence Type 15 Clone from Clinical Infections in Bangladesh

**DOI:** 10.1128/mSphere.00023-20

**Published:** 2020-03-11

**Authors:** Refath Farzana, Lim S. Jones, Andrew Barratt, Muhammad Anisur Rahman, Kirsty Sands, Edward Portal, Ian Boostrom, Laura Espina, Monira Pervin, A. K. M. Nasir Uddin, Timothy R. Walsh

**Affiliations:** a Department of Medical Microbiology, Institute of Infection and Immunity, School of Medicine, Cardiff University, Cardiff, United Kingdom; b Public Health Wales Microbiology, University Hospital of Wales, Cardiff, United Kingdom; c Department of Virology, Dhaka Medical College, Dhaka, Bangladesh; d Dhaka Medical College Hospital, Dhaka, Bangladesh; Escola Paulista de Medicina/Universidade Federal de São Paulo

**Keywords:** *mcr-8.1*, *Klebsiella pneumoniae*, human, Bangladesh

## Abstract

The emergence of mobilized colistin resistance genes (*mcr*) has become a serious concern in clinical practice, compromising treatment options for life-threatening infections. In this study, colistin-resistant Klebsiella pneumoniae harboring *mcr-8.1* was recovered from infected patients in the largest public hospital of Bangladesh, with a prevalence of 0.3% (3/1,097). We found *mcr-8.1* in an identical highly stable multidrug-resistant IncFIB(pQil) plasmid of ∼113 kb, which belonged to an epidemiologically successful K. pneumoniae clone, ST15. The resistance mechanism was proven to be horizontally transferable, which incurred a fitness cost to the host. The core genome phylogeny suggested the clonal spread of *mcr-8.1* in a Bangladeshi hospital. Core genome single-nucleotide polymorphisms among the *mcr-8.1*-positive K. pneumoniae isolates ranged from 23 to 110. It has been hypothesized that *mcr-8.1* was inserted into IncFIB(pQil) with preexisting resistance loci, *bla*_TEM-1b_ and *bla*_CTX-M-15_, by IS*903B*. Coincidentally, all resistance determinants in the plasmid [*mcr-8.1*, *ampC*, *sul2*, *1d-APH(6)*, *APH(3′′)-Ib*, *bla*_TEM-1b_, *bla*_CTX-M-15_] were bracketed by IS*903B*, demonstrating the possibility of intra- and interspecies and intra- and intergenus transposition of entire resistance loci. This is the first report of an *mcr*-like mechanism from human infections in Bangladesh. However, given the acquisition of *mcr-8.1* by a sable conjugative plasmid in a successful high-risk clone of K. pneumoniae ST15, there is a serious risk of dissemination of *mcr-8.1* in Bangladesh from 2017 onwards.

**IMPORTANCE** There is a marked paucity in our understanding of the epidemiology of colistin-resistant bacterial pathogens in South Asia. A report by Davies and Walsh (Lancet Infect Dis 18:256–257, https://doi.org/10.1016/S1473-3099(18)30072-0, 2018) suggests the export of colistin from China to India, Vietnam, and South Korea in 2016 was approximately 1,000 tons and mainly used as a poultry feed additive. A few reports forecast that the prevalence of *mcr* in humans and livestock will increase in South Asia. Given the high prevalence of *bla*_CTX-M-15_ and *bla*_NDM_ in India, Bangladesh, and Pakistan, colistin has become the invariable option for the management of serious infections, leading to the emergence of *mcr*-like mechanisms in South Asia. Systematic scrutiny of the prevalence and transmission of *mcr* variants in South Asia is vital to understanding the drivers of *mcr* genes and to initiate interventions to overcome colistin resistance.

## Abstract (precis)

There is a marked paucity in our understanding of the epidemiology of colistin-resistant bacterial pathogens in South Asia. A report by Davies and Walsh (Lancet Infect Dis 18:256–257, https://doi.org/10.1016/S1473-3099(18)30072-0, 2018) suggests the export of colistin from China to India, Vietnam, and South Korea in 2016 was approximately 1,000 tons and mainly used as a poultry feed additive. A few reports forecast that the prevalence of *mcr* in humans and livestock will increase in South Asia. Given the high prevalence of *bla*_CTX-M-15_ and *bla*_NDM_ in India, Bangladesh, and Pakistan, colistin has become the invariable option for the management of serious infections, leading to the emergence of *mcr*-like mechanisms in South Asia. Systematic scrutiny of the prevalence and transmission of *mcr* variants in South Asia is vital to understanding the drivers of *mcr* genes and to initiate interventions to overcome colistin resistance.

## OBSERVATION

Klebsiella pneumoniae is an opportunistic Gram-negative pathogen that is mostly associated with nosocomial infections (1). Carbapenems have been widely used to treat multidrug-resistant (MDR) K. pneumoniae infections, leading to the emergence of carbapenem resistance, where colistin is one of a few viable options (2, 3). The prevalence of colistin resistance is rapidly expanding in South Asia (4–10). Colistin resistance is either mediated by mutational disruption or insertional inactivation of *mgrB* (11) or via the acquisition of MCR plasmid-mediated resistance (12, 13). Here, we characterize a clinical epidemic K. pneumoniae clone harboring *mcr-8.1* from a Bangladeshi hospital.

A pilot antimicrobial resistance (AMR) survey was conducted from 21 October 2016 to 23 September 2017 at Dhaka Medical College Hospital (DMCH), which included 1,097 culture-positive clinical specimens. The project was approved by the Ethical Review Committee of DMCH (MEU-DMC/ECC/2017122). K. pneumoniae was recovered on chromogenic UTI containing vancomycin (10 mg/liter) and identification by matrix-assisted laser desorption ionization–time of flight mass spectrometry (MALDI-TOF MS; MALDI Biotyper; Bruker Daltonics, Inc., Billerica, MA, USA). MICs of relevant antimicrobials were determined by agar dilution and the MIC of colistin by broth microdilution. Susceptibility patterns of antimicrobials were interpreted according to EUCAST breakpoints. Sequencing was performed using Illumina MiSeq (Illumina Inc., San Diego, CA) and Nanopore (Oxford Nanopore Technologies, Oxford, UK) platforms. We adopted a hybrid strategy to assemble draft genomes using Unicycler (v0.4.0) (see Text S1 in the supplemental material). Pangenome analysis was performed using Roary (v3.12.0). A maximum likelihood phylogenetic tree was built using FastTree (v2.1.0) and visualized using Phandango and iTOL (v5.3). Intraclade single-nucleotide polymorphisms (SNPs) were identified using Snippy (v4.4.5). Plasmid size was confirmed by pulsed-field gel electrophoresis (PFGE) of S1 nuclease DNA digests and *mcr-8.1* probing. Conjugation assays were performed using Escherichia coli J53 as the recipient (9). Serial passaging of MCR-positive K. pneumoniae (MCRPKP) was performed in a colistin-free medium up to 12 days, and genomic DNA (gDNA) was extracted on days 0, 3, 6, 9, and 12. Plasmid stability was assessed by relative abundance of *mcr-8.1* compared to that of housekeeping genes (HKGs) using quantitative PCR (Bio-Rad, USA) (Text S2). The *in vitro* growth rate of E. coli J53 and transconjugants (TDM697b, TDM782, and TDM914b) was determined by optical density (OD) in 30-min intervals for 24 h using FLUOstar Omega (BMG Labtech Ltd., Aylesbury, UK). The growth rate of each transconjugant was compared to that of E. coli J53 by unpaired two-tailed *t* test using GraphPad Prism (v7.04) (Text S3).

10.1128/mSphere.00023-20.6 TEXT S1 Illumina MiSeq sequencing and bioinformatics, MinION sequencing and bioinformatics, core genome phylogenetic analysis, and *in silico* genome-wide analysis. Download Text S1, DOCX file, 0.02 MB. Copyright © 2020 Farzana et al. 2020 Farzana et al. This content is distributed under the terms of the Creative Commons Attribution 4.0 International license.

10.1128/mSphere.00023-20.7 TEXT S2 Stability of plasmid carrying MCR-8.1 in K. pneumoniae. Download Text S2, DOCX file, 0.02 MB. Copyright © 2020 Farzana et al. 2020 Farzana et al. This content is distributed under the terms of the Creative Commons Attribution 4.0 International license.

10.1128/mSphere.00023-20.8 TEXT S3 *In vitro* time-growth studies. Download Text S3, DOCX file, 0.02 MB. Copyright © 2020 Farzana et al. 2020 Farzana et al. This content is distributed under the terms of the Creative Commons Attribution 4.0 International license.

## 

### Description of cases with infections by MCRPKP.

In this study, 3 K. pneumoniae isolates (3/1,097, 0.3%) were phenotypically resistant to colistin. MCRPKP isolates were recovered from the urine of two patients admitted under urology and the blood of a third patient in the neonatal intensive care unit (NICU). Case 1 (BD_DM_697) was a 55-year-old male with benign enlargement of the prostate with diabetes mellitus and a history of catheterization for 13 days. Case 2 (BD_DM_782) was a 63-year-old male patient with a left renal tumor, a history of catheterization for 15 days, and hematuria. These patients were discharged on days 20 and 35 of hospitalization, respectively. Case 3 (BD_DM_914) was a 5-day preterm low-birth-weight neonate with late-onset neonatal sepsis who died within 18 days after hospital admission. We did not observe any overlapping of hospital stay among the MCRPKP cases. MCRPKP isolates were coresistant to amoxicillin-clavulanate, piperacillin-tazobactam, cephalosporins (ceftazidime and cefotaxime), ciprofloxacin, levofloxacin, gentamicin, trimethoprim-sulfamethoxazole, and colistin and susceptible to carbapenems, amikacin, fosfomycin, and tigecycline. Although MCRPKP cases initially were shown to be treated with inappropriate antimicrobials, we have no data on whether the antibiotic therapy was subsequently changed based on the sensitivity report from the local laboratory.

### Clonal spread of *mcr-8.1*.

**FIG 1:**
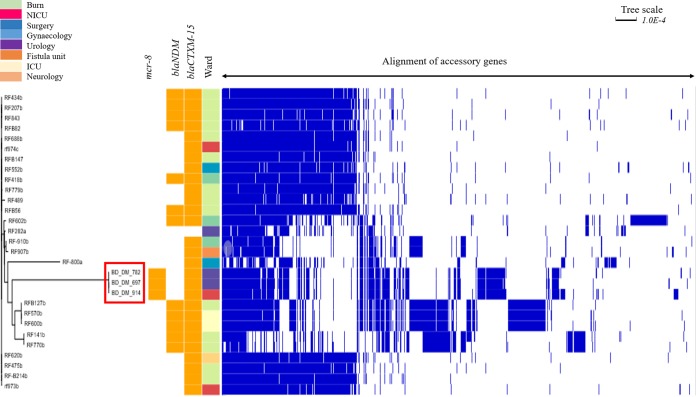
Phylogenetic tree of K. pneumoniae ST15 identified in this study (*n* = 29). Shown is a maximum likelihood (ML) phylogenetic tree constructed using a pangenome alignment. Strains were grouped together based on the similarity of genes and the presence of genes in the accessory genome using Roary (v3.12.0). Epidemiologically important resistance genes are indicated by orange cells, accessory genes by blue cells, and the absence of genes by white cells. NICU, neonatal intensive care unit; ICU, intensive care unit.

**FIG 2:**
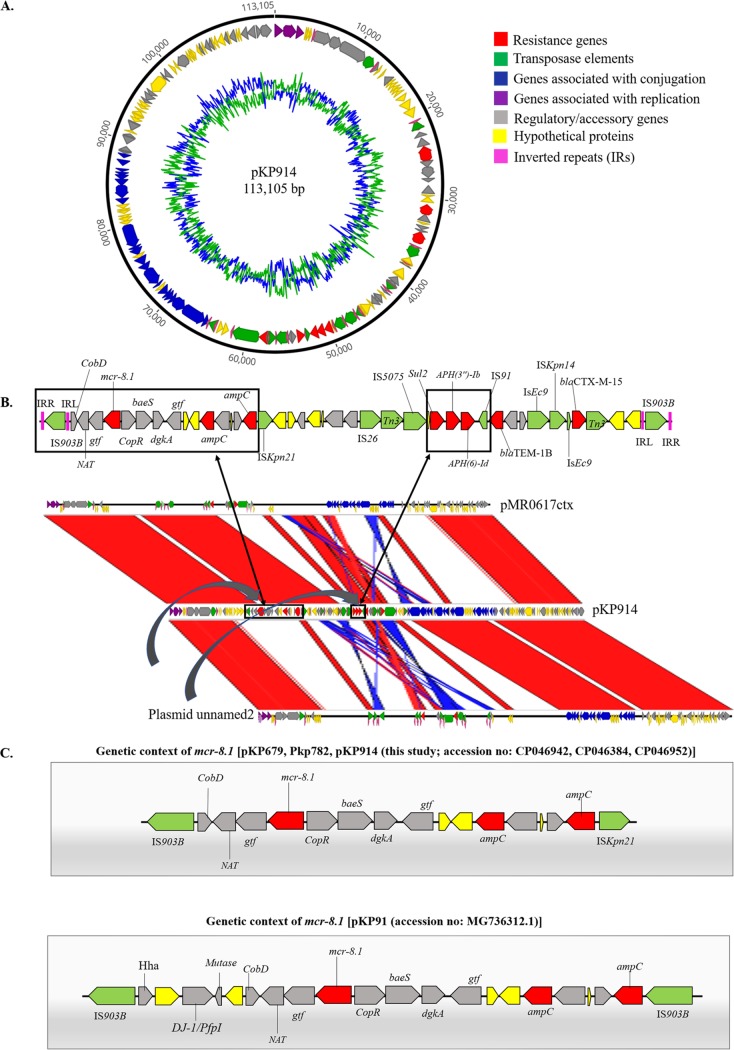
Genetic organization of plasmid harboring *mcr-8.1*. (A) Circular view of pKP914 (accession no. CP046952). (B) Schematic layout of sequence comparison of pKP914 (accession no. CP046952) against FDAARGOS_440 plasmid unnamed2 (accession no. CP023922.1) and pMR0617ctx (accession no. CP024040.1). Arrows represent the position and transcriptional direction of the open reading frames. Genomic comparison was performed by Artemis Comparison Tool (ACT) (v.18.0.1). (C) Comparison of genetic environments of *mcr-8.1*. *APH*, aminoglycoside phosphotransferase; *baeS*, histidine-protein kinase; *bla*, beta-lactamase; *Cob*, cobalamin biosynthesis; *Cop*, copper homeostasis transcription factor; *dgkA*, diacylglycerol kinase; *DJ-1/PfpI*, cysteine peptidase; *gtf*, glucosyltransferase; Hha, hemolysin expression-modulating protein; IRL, inverted repeat left; IRR, inverted repeat right; IS, insertion sequence; *mcr-8.1*, mobilized colistin resistance*; NAT*, *N*-acetyltransferase; *Sul2*, dihydropteroate synthase.

*In silico* genome-wide analysis of MCRPKP detected a 1,698-bp open reading frame (ORF), encoding a phosphoethanolamine transferase, showing 100% nucleotide identity to *mcr-8.1*. The prevalence of K. pneumoniae among all clinical isolates from this study was 21% (228/1097), of which 13% (29/228) belonged to ST15. K. pneumoniae ST15 harboring *mcr-8.1* was clustered in one clade (Fig. 1), suggesting the clonal spread of MCRPKP. SNP mapping found 110 and 107 SNPs in BD_DM_782 and BD_DM_914, respectively, compared to BD_DM_697, and 23 SNPs in BD_DM_782 compared to BD_DM_914. MCR-8 was described previously in K. pneumoniae ST1, of human origin, and K. pneumoniae ST42, of animal origin (14). K. pneumoniae ST15 has been regarded as a successful clone in disseminating *bla*_CTX-M-15_ globally (15). The draft genome sequences and S1 PFGE indicate that *mcr-8.1* elements in K. pneumoniae were located on identical IncFIB(pQil) plasmids of ∼113 kb (GenBank accession no. CP046384, CP046952, and CP046942) (Fig. 2 and Fig. S1). The gene *mcr-8.1* was stable after serial passaging without any antibiotic challenge. Compared to that at day 0, the abundance of *mcr-8.1* versus HKG was static up to day 12 (Fig. S2). Yang et al. (16) reported that colistin susceptibility could be attenuated after serial passaging of *mcr-1*-positive strains in antibiotic-free medium. Our findings demonstrate that the IncFIB(pQil) plasmid harboring *mcr-8.1* was remarkably stable, suggesting adaptive plasmid-host evolution (17). K. pneumoniae ST15 can be a vector capable of spreading *mcr*-mediated colistin resistance, particularly in a setting with suboptimal infection control practices (18).

10.1128/mSphere.00023-20.1 FIG S1 Pulsed-field gel of S1 nuclease-digested gDNA carrying *mcr-8.1* and in-gel hybridization with *mcr-8.1* probe. Download FIG S1, TIF file, 0.9 MB. Copyright © 2020 Farzana et al. 2020 Farzana et al. This content is distributed under the terms of the Creative Commons Attribution 4.0 International license.

10.1128/mSphere.00023-20.2 FIG S2 Stability of plasmid-mediated colistin resistance in K. pneumoniae. Colistin resistance mechanism *mcr-8.1* was highly stable after 12 days of passaging in colistin-free medium. Download FIG S2, TIF file, 0.1 MB. Copyright © 2020 Farzana et al. 2020 Farzana et al. This content is distributed under the terms of the Creative Commons Attribution 4.0 International license.

### Genetic context and dynamics of plasmids harboring *mcr-8.1*.

Genome-wide analyses demonstrated that the plasmids recovered from the MCRPKP were almost identical to each other (Fig. S3). Complete plasmid sequences were determined for pKP782 (accession no. CP046384) and pKP914 (accession no. CP046952) by hybrid assembly, while pKP697 (accession no. CP046942) was not successfully closed. One copy of the IncFIB(pQil) plasmid with an identical resistance profile was recovered from each MCRPKP isolate and shared 99.72% nucleotide identity at 70% coverage with previously described plasmids (accession no. CP023922.1 and CP024040.1). However, those plasmids (CP023922.1 and CP024040.1) were absent from *mcr*-like genes (Fig. 2B). The genetic environment around *mcr-8.1* in IncFIB(pQil)-MCR-8.1 (pKP697, pKP782, and pKP914) shares identity with a previously described *mcr-8.1*-containing plasmid isolated from pigs in China (accession no. MG736312.1), although the plasmids harboring *mcr-8.1* in this study are truncated at the 5′ end and IS*903B* at the 3′ end was replaced by ISKpN14 (14) (Fig. 2C). It is possible that *mcr-8.1* originally was transposed to the IncFIB(pQil) plasmid by an IS*903B* composite transposon (Fig. 2B and C). An array of AMR genes (*bla*_TEM-1b_ and *bla*_CTX-M-15_) was in a composite transposon, flanked by insertion sequences (Fig. 2B). Incidentally, all resistance components in IncFIB(pQil) plasmids in this study were bracketed by IS*903B* from nucleotide position 20590 to 64656, demonstrating the potential for the transposition of the entire intervening DNA segment. The conjugation assay confirmed the transferability of the plasmid containing *mcr-8.1* to E. coli J53 with a frequency range of 3.1 × 10^−2^ to 8 × 10^−2^. Phenotypically, the transconjugants were resistant to ampicillin, amoxicillin-clavulanate, 3rd-generation cephalosporins, trimethoprim-sulfamethoxazole, and colistin (Table S1).

10.1128/mSphere.00023-20.3 FIG S3 Linear comparison of plasmid sequence pKP914 (accession no. CP046952) and pKP782 (accession no. CP046384), recovered in this study. Forward matches are indicated by red and reverse matches by blue. Regions of sequence with homology to the other genomes are separated by black lines. Genomic comparison was performed by Artemis Comparison Tool (ACT) (v.18.0.1). Download FIG S3, TIF file, 0.4 MB. Copyright © 2020 Farzana et al. 2020 Farzana et al. This content is distributed under the terms of the Creative Commons Attribution 4.0 International license.

10.1128/mSphere.00023-20.5 TABLE S1 Range of MIC values of transconjugants obtained in this study along with donors and recipient. Download Table S1, DOCX file, 0.01 MB. Copyright © 2020 Farzana et al. 2020 Farzana et al. This content is distributed under the terms of the Creative Commons Attribution 4.0 International license.

The acquisition of a resistance plasmid may impose a fitness cost, depending on the host and plasmid backbones (19, 20). We found a significantly lower growth rate over time in TDM697b and TDM914b relative to that of E. coli J53 (*P* < 0.0001) (Fig. S4), implying a significant fitness cost owing to the acquisition of a plasmid harboring *mcr-8.1*. Compared to that of E. coli J53, a lower growth rate was also observed in TDM782b; however, the fitness cost was not statistically significant (Fig. S4).

10.1128/mSphere.00023-20.4 FIG S4 Analysis of bacterial fitness cost. (A) Growth curve of transconjugants compared to that of E. coli J53. (B) Growth rate of transconjugants compared to that of E. coli J53, fitted with the Gompertz model. Graphs show the average value from five replicates ± standard deviations (or standard errors of the means). Download FIG S4, TIF file, 0.3 MB. Copyright © 2020 Farzana et al. 2020 Farzana et al. This content is distributed under the terms of the Creative Commons Attribution 4.0 International license.

This is the first report of transferable colistin resistance associated with human infections from Bangladesh. Given the acquisition of *mcr-8.1* on a conjugative plasmid, with good stability in ST15, a successful high-risk clone of K. pneumoniae, there is a serious risk of dissemination of *mcr-8.1* in South Asia.

### Accession number(s).

The nucleotide sequences of MCRPKP isolates are available under NCBI accession no. CP046939 to CP046947, CP046381 to CP046385, and CP046939 to CP046947.
