# Supplementary material for: Emergence of Mobile Colistin Resistance (mcr-8) in a Highly Successful Klebsiella pneumoniae Sequence Type 15 Clone from Clinical Infections in Bangladesh
Source: mSphere. 2020 Mar 11;5(2):e00023-20. doi: 10.1128/mSphere.00023-20 (PMC7067589; doi:10.1128/mSphere.00023-20)
Supplement: TEXT S1 [file mSphere.00023-20-s0001.docx]

**Illumina MiSeq sequencing and bioinformatics**

Whole genome sequencing (WGS) was performed on the Illumina MiSeq platform (Illumina Inc., San Diego, CA). Briefly, gDNA was extracted from overnight culture using the QIAcube (Qiagen, Hilden, Germany), and resulting gDNA was quantified using the Qubit 3.0 (Thermos Fisher Scientific, Waltham, USA). DNA libraries were prepared for paired end sequencing (2x301 cycles) using Nextera XT. Quality control of raw reads included fastqc (0.11.2), and quality and adaptor trimming was performed using Trimgalore (0.4.3). Reads were assembled in contigs using the *de novo* assembler SPAdes (3.9.0) (.fasta) and were aligned to the original fastq reads using BWA aligner (0.7.15). Any error was corrected using Pilon (1.22). Assembly metrics were evaluated using Quast (2.1).

**MinION sequencing and bioinformatics**

MCR-8 positive strain was sequenced by MinION sequencing (Oxford Nanopore Technologies Ltd., Oxford Science Park, UK). For this sequencing platform, gDNA was extracted using the QIAcube (Qiagen, Hilden, Germany) and DNA was processed using AMPure XP (Beckman Coulter, California, USA) to aim a concentration 53.3 ng/µl. DNA library was prepared by pooling of all barcoded samples and 1 µl of RAD was added to DNA. A final mixture of 75 µl (34 µl sequencing buffer, 30 µl water and 11 µl DNA library) was loaded to flow cell. MinION device was connected to MinKNOW GUI to obtain the reads. Demultiplexing of Nanopore reads was performed by Porechop (0.2.3). Unicycler (0.4.4) was used to yield hybrid assembly using both Illumina short reads and minION long reads.

**Core genome phylogenetic analysis**

The *de novo* assembly produced multiple contigs (145-330), with a mean GC content of 50%, annotated with Prokka (v1.12). Annotated assemblies generated by Prokka were used for large-scale pan-genome analysis using Roary (v3.12.0). Isolates were clustered according to the similarity of genes and presence of genes in the accessory genome. We constructed a maximum likelihood (ML) tree with FastTree (2.1.3) using general time reversible (GTR) evolutionary model and CAT approximation. To estimate the support for each split, we used 1,000 resamples (by default). FastTree computes [local support values](http://www.microbesonline.org/fasttree/#Support) with the [Shimodaira-Hasegawa test](http://mbe.oxfordjournals.org/cgi/reprint/16/8/1114). The ML tree was visualized using Phandango and iTOL (v5.3). Intra-clade SNPs were identified using Snippy (v4.4.5).

***In silico* genome-wide analysis**

Query sequences were searched for matching with similar sequenes in National Center for Biotechnology Information (NCBI) database using Basic Local Alignment Search Tool (BLAST) (https://blast.ncbi.nlm.nih.gov). Resistance genes were retrieved using ResFinder, plasmid replicons using PlasmidFinder and multilocus sequence typing (MLST) (7 loci) using MLST database in the Center for Genomic Epidemiology (CGE) (https://cge.cbs.dtu.dk). Insertion sequences (IS) were identified using ISfinder (https://isfinder.biotoul.fr).
