# Supplementary material for: Emergence of Mobile Colistin Resistance (mcr-8) in a Highly Successful Klebsiella pneumoniae Sequence Type 15 Clone from Clinical Infections in Bangladesh
Source: mSphere. 2020 Mar 11;5(2):e00023-20. doi: 10.1128/mSphere.00023-20 (PMC7067589; doi:10.1128/mSphere.00023-20)
Supplement: TEXT S2 [file mSphere.00023-20-s0002.docx]

### **Stability of plasmid carrying MCR-8.1 in *K. pneumoniae***

Serial passaging of *mcr-8.1* positive *K. pneumoniae* (MCRPKP) was performed in a colistin-free medium up to 12 days to investigate whether the *mcr-8* was stable. Overnight cultures of MCRPKP were diluted 1:1,000 in fresh LB medium without colistin and incubated with vigorous shaking (220 rpm) at 37°C for 24 h. Total gDNA was extracted on day 0, day 3, day 6, day 9 and day 12 using the QIAcube (Qiagen, Hilden, Germany) from the overnight culture with optical density (OD) ranged from 0.08 to 0.1 at 600 nm. OD value was measured using Jeneway 7315 spectrophotometer (Geneflow limited, Staffordshire, UK). The prepared DNA was analysed to quantify *mcr-8.1* (plasmid-borne) and *RcsA*, a housekeeping gene (HKG) in triplicate by real-time quantitative PCR (qPCR). Primers and probes were designed manually using the Geneious prime primer design tool (11.0.2; Biomatters Ltd.) and synthesised by Eurofins (Ebersberg, Germany). The primers and probes used in this study are shown the Table below. The real-time qPCR mixture of 20 µl was prepared using 10 µl SSO advanced master mix (Bio-Rad, USA), 0.4 µl primer mix (mixture of 10 µL of each primer [100 pmol] and 60 µl of molecular grade water), 0.4 µl probe mix (mixture of 10 µL of each probe [100 pmol] and 80 µl of molecular grade water), 4.2 µl of molecular grade water and 5 µl template DNA. qPCR was performed using the CFX96 real-time system (Bio-Rad, USA) with cycling parameters of 1 x (95°C x 5 minutes), 44 x (95°C x 15 seconds, 60°C x 10 second) and *C*_T_ value was measured by maestro interpretative software installed in the qPCR system. Relative abundance of *mcr-8.1*, compared to HKG were calculating by delta-delta *C*_T_ method (2^–∆∆Ct^). Gene expression on day 0 was used a control.

**Table Sequences of primers and probes for qPCR designed in this study**

| Name | Target | Tm | GC% | Sequence | Product length (bp) | Product (Tm) |
| --- | --- | --- | --- | --- | --- | --- |
| MCR 8_F | MCR8 | 58.2 | 52.0 | CTCGCTTGCAGATTCCCTTACAACC | - | - |
| MCR 8_R | MCR8 | 58.2 | 52.2 | TCCGTGCCGCATCAGAAGAAAAC | - | - |
| MCR 8_ Probe | MCR8 | 61.7 | 46.7 | ACCATTGTTATTGTTGCGCCATAGCACCTC | 108 | 73.0 |
| RcsA_F | *K. pneumoniae* | 58.4 | 59.1 | TCCCAACCGGGTATAGCTGCAC | - | - |
| RcsA_R | *K. pneumoniae* | 59.3 | 59.1 | TATCGCCCGCAAGGACTGCTTC | - | - |
| RcsA_Probe | *K. pneumoniae* | 63.1 | 41.0 | ACATGACCCATCCTCAATCAACACGTAACGATATACACT | 117 | 74.5 |
