# Supplementary material for: Emergence of Mobile Colistin Resistance (mcr-8) in a Highly Successful Klebsiella pneumoniae Sequence Type 15 Clone from Clinical Infections in Bangladesh
Source: mSphere. 2020 Mar 11;5(2):e00023-20. doi: 10.1128/mSphere.00023-20 (PMC7067589; doi:10.1128/mSphere.00023-20)
Supplement: TEXT S3 [file mSphere.00023-20-s0003.docx]

***In vitro* time-growth studies**

## Bacterial suspension of optical density (OD) ranged from 0.08 to 0.1 at 600 nm was prepared from overnight culture and diluted 1:100 in fresh LB medium. *In vitro* growth rate of *E. coli* J53 and transconjugants (TDM_697b, TDM_782 and TDM_914b) was determined by OD in 30 minutes interval for 24 hours with shaking at 100 rpm using FLUOstar Omega microplate reader (BMG LABTECH Ltd., Aylesbury, UK). The experiment was performed in five replicates. The growth curve of each strain was generated with mean OD at each time point and was fitted to the modified Gompertz model developed by Zwietering et al. (1990) using GraphPad Prism (v7.04) (1). The growth rate and lag time were calculated. The growth rate of each transconjugant was compared to that of *E. coli* J53 by unpaired two-tailed t test using GraphPad Prism (v7.04) (TEXT S3).

## **REFERENCES**

## Zwietering MH, Jongenburger I, Rombouts FM, van 't Riet K. 1990. Modeling of the bacterial growth curve. Appl Environ Microbiol 56:1875-81.
