# Supplementary figures and images for: Emergence of Mobile Colistin Resistance (mcr-8) in a Highly Successful Klebsiella pneumoniae Sequence Type 15 Clone from Clinical Infections in Bangladesh
Source: mSphere. 2020 Mar 11;5(2):e00023-20. doi: 10.1128/mSphere.00023-20 (PMC7067589; doi:10.1128/mSphere.00023-20)

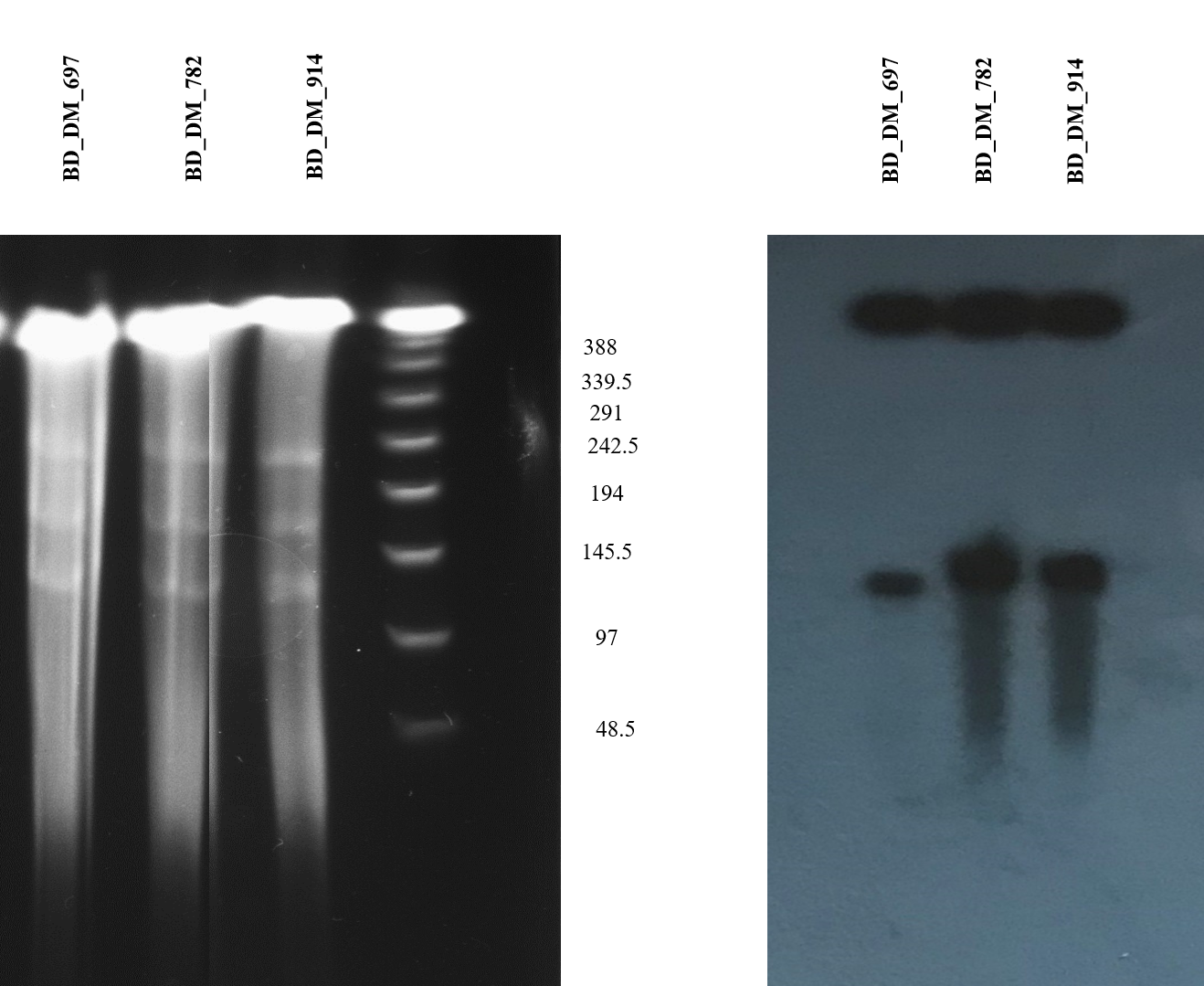

Supplement: FIG S1 [file mSphere.00023-20-sf001.tif]

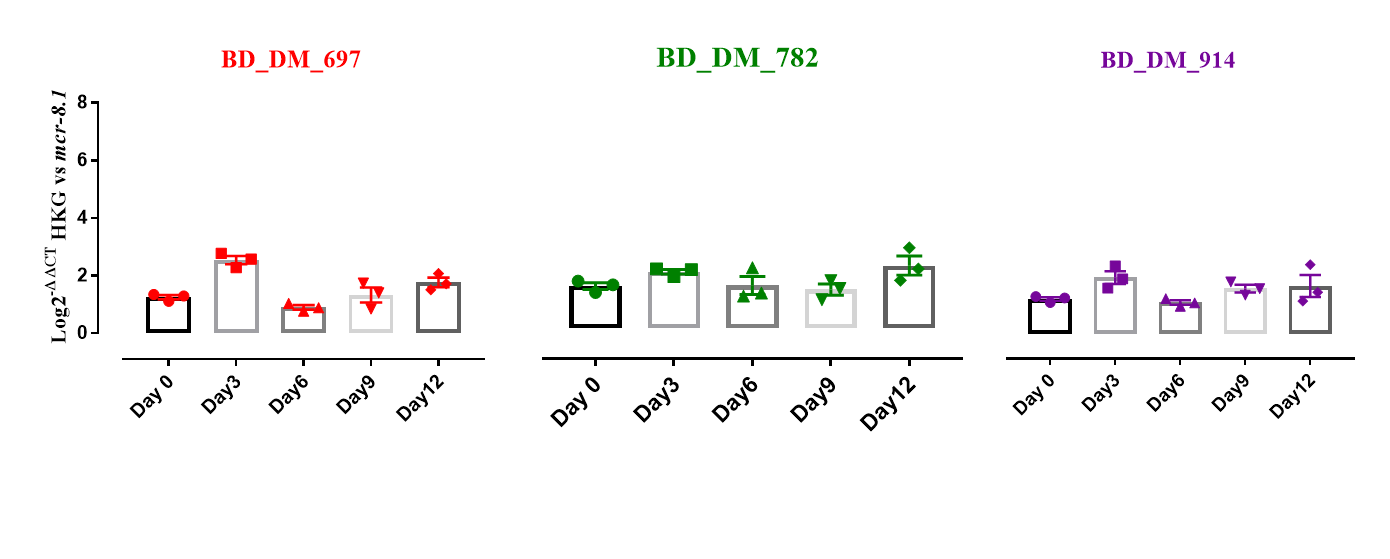

Supplement: FIG S2 [file mSphere.00023-20-sf002.tif]

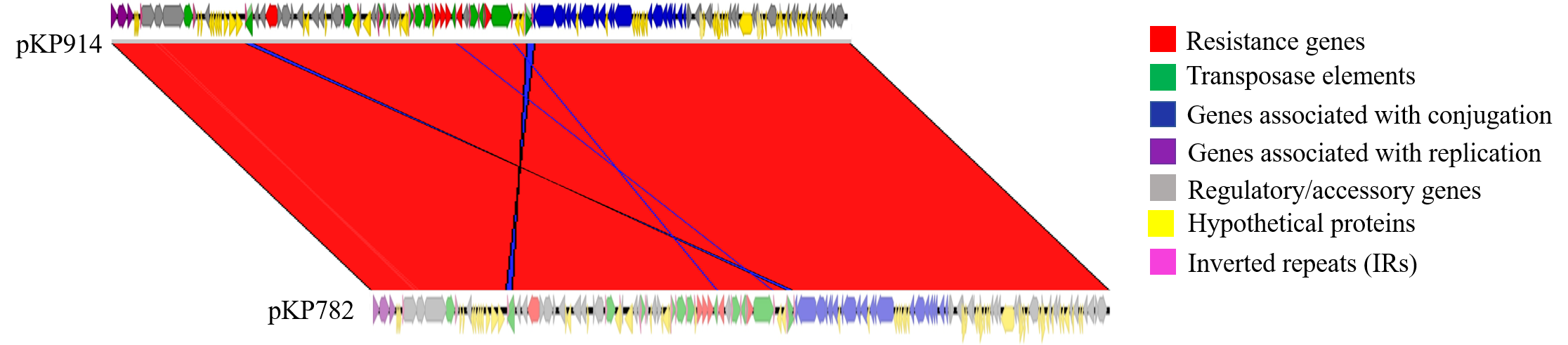

Supplement: FIG S3 [file mSphere.00023-20-sf003.tif]

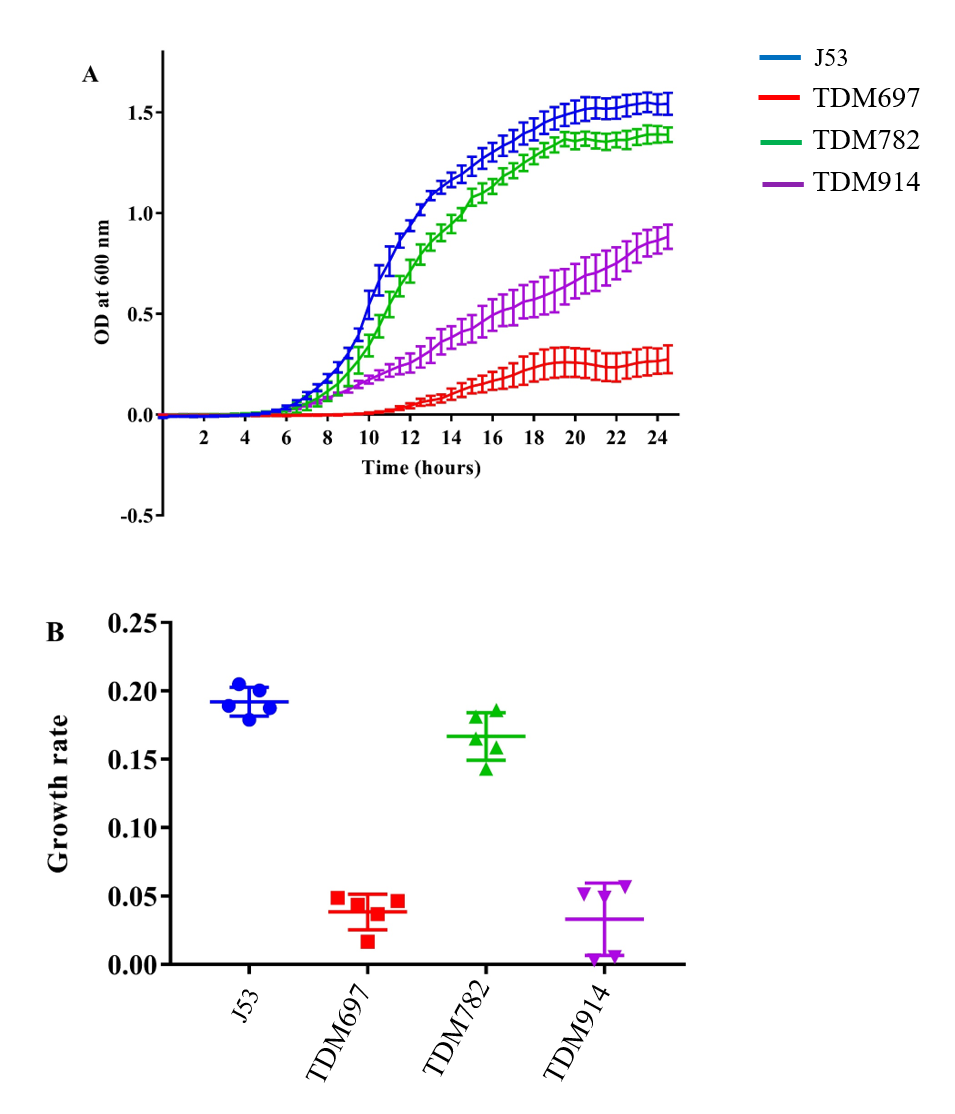

Supplement: FIG S4 [file mSphere.00023-20-sf004.tif]
