# Supplementary material for: Emergence of Mobile Colistin Resistance (mcr-8) in a Highly Successful Klebsiella pneumoniae Sequence Type 15 Clone from Clinical Infections in Bangladesh
Source: mSphere. 2020 Mar 11;5(2):e00023-20. doi: 10.1128/mSphere.00023-20 (PMC7067589; doi:10.1128/mSphere.00023-20)
Supplement: TABLE S1 [file mSphere.00023-20-st001.docx]

**Table S1 Range of MIC values of transconjugants obtained in this study along with donors and recipient**

| Antibiotics | Donors | Recipient (*E. coli* J53) | Transconjugants |
| --- | --- | --- | --- |
| Ampicillin | 128-≥256 | 2 | 64-256 |
| Amoxicillin-clavulanate | 256 | 2 | 64-256 |
| Piperacillin-tazobactam | 16 | 2 | 2 |
| Ceftazidime | 32 | ≤0.06 | 32 |
| Cefotaxime | 128 | ≤0.06 | 128 |
| Cefepime | 16-32 | ≤0.06 | 0.25 |
| Imipenem | 0.25 | ≤0.06 | ≤0.06 |
| Meropenem | ≤0.06 | ≤0.06 | ≤0.06 |
| Ciprofloxacin | 256 | ≤0.06 | ≤0.06 |
| Levofloxacin | 64 | ≤0.06 | ≤0.06 |
| Amikacin | 2-4 | 2 | 2 |
| Gentamicin | 32 | 0.5 | 0.5 |
| Trimethoprim-sulfamethoxazole | 128 | 2 | 32-128 |
| Colistin | 8 | ≤0.06 | 8 |
